# Tailored Biochar–*Pseudomonas chlororaphis* Composites for Triclocarban Removal: A Feedstock-Dependent Structure–Interface–Metabolism Study

**DOI:** 10.3390/ijms27062684

**Published:** 2026-03-15

**Authors:** Changlei Wang, Chongshu Li, Fangrong Wei, Jialin Liu, Yan Long, Jinshao Ye

**Affiliations:** School of Environment and Climate, Jinan University, Guangzhou 510630, China

**Keywords:** biochar, *Pseudomonas chlororaphis*, extracellular electron transfer, metabolomics, triclocarban

## Abstract

Biochar provides a porous scaffold, conductive carbon framework and redox-active surface functional that can promote microbial attachment and extracellular electron flow. However, how feedstock-dependent biochar properties regulate the biochar–cell interface and microbial metabolism during contaminant removal remains insufficiently understood. Here, biochar derived from rice husk, corn straw and corn cob was used to immobilize *Pseudomonas chlororaphis* for triclocarban removal in batch microcosms. Multiscale analyses, including scanning electron microscopy (SEM), fourier transform infrared spectroscopy (FTIR), X-ray photoelectron spectroscopy (XPS), cyclic voltammetry (CV), (electrochemical impedance spectroscopy (EIS) and liquid chromatography–mass spectrometryLC-MS, were combined to link the biochar structure, interface and extracellular metabolism signatures with triclocarban (TCC) removal. Compared with free cells, all composites enhanced TCC removal and exhibited altered interfacial functional-group features together with substantially reduced fitted charge-transfer resistance, indicating facilitated interfacial electron exchange. Untargeted metabolomics further revealed consistent remodeling of extracellular redox-associated metabolite signatures upon immobilization, with increased quinone/polyphenol-associated features and pathway-level shifts related to redox homeostasis. Among feedstocks, the corn cob composite showed the highest triclocarban removal. Overall, this work proposes an evidence-supported “structure–interface–metabolism” framework for interpreting how agricultural-residue biochars modulate biofilm interfaces and redox-related metabolic signatures to improve triclocarban removal, providing guidance for designing biochar-supported bioprocesses for halogenated micropollutants.

## 1. Introduction

Biochar–bacteria composites (BBCs) have emerged as a promising approach for pollution remediation [1]. Applications of BBCs have already been reported in the United States and Europe for the removal of hydrocarbons and chlorinated organics, where stable biofilms contribute to high degradation efficiency [2,3]. In Asia, research has shown that biochar enhances the degradation of antibiotics and pesticides through redox-active oxygen-containing groups [4]. Biochar possesses a large surface area, hierarchical porosity, and abundant surface functional groups such as quinones, phenols, carboxyls, and amines [5]. These features offer stable sites for microbial colonization, create protective microhabitats, and participate directly in electron exchange [6]. These surface moieties are also mechanistically relevant to EET: quinone-/phenolic-like groups can participate in reversible redox reactions and thereby facilitate mediated/interfacial electron exchange [7], whereas oxygenated groups (e.g., carboxyls/hydroxyls) can tune hydrophilicity and adhesion, indirectly affecting biofilm formation and interfacial resistance [8]. In parallel, the degree of aromaticity/graphitization and heteroatom doping can influence conductivity and thus the contribution of more direct electron-transfer pathways. When microorganisms are immobilized on biochar, the interfacial resistance of the composite is reduced and diffusion pathways are shortened, leading to enhanced extracellular electron transfer (EET) and reshaped microbial metabolic pathways [9]. Consequently, BBCs can achieve faster and more efficient pollutant degradation than free-living cells [10,11,12].

Triclocarban (TCC), a halogenated antibacterial agent widely used in personal care products, is frequently detected in surface water, sediments, and even drinking water [13,14]. Its chlorinated aromatic structure renders it highly persistent and poorly degradable, posing ecological and health risks [15,16]. Conventional wastewater treatment methods fail to remove it effectively, and some degradation byproducts are even more toxic [17,18]. Thus, TCC serves as an ideal probe compound for studying the degradation of halogenated persistent organic pollutants (POPs) and contaminants of emerging concern (CECs). Developing biochar-assisted bioprocesses for TCC removal is therefore of considerable interest for upgrading conventional wastewater treatment plants. *Pseudomonas chlororaphis* (*P. chlororaphis*) is a versatile bacterium capable of degrading aromatic and chlorinated compounds via efficient EET processes [19,20], making it a suitable model organism for BBCs targeting TCC.

In this study, an initial TCC concentration of 1 mg/L was employed as a deliberately elevated, yet realistic, high-load level. This concentration falls within the upper range occasionally reported for industrial and hospital effluents and accident-related releases, and was selected both to simulate extreme loading conditions and to ensure a sufficient analytical resolution for elucidating degradation mechanisms in batch microcosms. Under such “stress-tested” conditions, differences in electron generation, transfer and regeneration among biochar–bacteria composites are amplified, facilitating the identification of feedstock-dependent structure–interface–metabolism relationships that remain informative for process design at lower, environmentally relevant TCC levels.

Biochars derived from agricultural wastes, including rice husk (RH), corn straw (CS) and corn cob (CC), are attractive low-cost carriers for constructing BBCs, because they are renewable materials with favorable conductivity and buffering capacity and can support microbial diversity and community stability within composites [21,22]. Importantly, these agricultural feedstocks differ in lignocellulosic composition and ash/mineral content, leading to distinct pore structures and surface chemistries after pyrolysis [23]. Accordingly, feedstock-dependent differences are typically reflected in quantifiable metrics such as (Brunauer–Emmett–Teller) BET surface area and pore volume, ash/mineral fractions (e.g., Si-rich residues in rice husk biochar), elemental ratios (H/C and O/C), and the relative abundance of key functional groups resolved by FTIR/XPS [7]. For example, corn straw typically yields biochar with a higher specific surface area and pore volume, whereas corn cob tends to produce biochar with more polar oxygen-containing functional groups and higher surface polarity. Rice husk biochar is characterized by a relatively high ash and silica content, forming a unique Si-rich porous framework. Such feedstock-dependent differences in porosity, hydrophilicity and functional-group distribution are expected to modulate bacterial attachment, interfacial electron exchange and metabolic responses in BBCs.

For halogenated aromatic contaminants such as TCC, biochar-based approaches have most commonly been framed around adsorptive sequestration, or around hybrid schemes that pair biochar with catalytic/oxidative or reductive processes to improve transformation. However, the extent to which the feedstock-derived surface chemistry and redox-active functional reshape microbial electron-transfer behavior and cellular redox metabolism during dechlorination remains insufficiently resolved. Despite accumulating evidence of the synergistic performance of BBCs, most previous studies have focused either on adsorption and overall degradation efficiency or on electrochemical characterization of biochar-based materials [24]. Only a few have systematically integrated microstructural, interfacial and metabolic information to elucidate how specific biochar properties, such as pore structure, surface polarity and oxygen-containing functional groups, coordinately shape bacterial EET pathways and metabolic adaptation [25]. In particular, the links between feedstock-dependent surface chemistry, quinone/polyphenol-mediated electron shuttling, and the reconfiguration of redox-related pathways (e.g., aromatic degradation, pentose phosphate pathway and glutathione metabolism) remain largely unexplored, especially for halogenated POPs such as TCC.

Although the present work is performed in well-controlled batch microcosms and focuses on mechanistic aspects of extracellular electron transfer and metabolic regulation, it is explicitly motivated by process engineering needs. Rather than aiming to reproduce full-scale operating conditions, we seek to establish how feedstock-dependent differences in biochar structure and surface chemistry translate into distinct electron-transfer pathways and metabolic strategies in *P. chlororaphis*. Such a process-oriented mechanistic understanding is essential for the rational selection and tailoring of biochar carriers in biofilm and bioelectrochemical reactors for the polishing of TCC and structurally related halogenated micropollutants.

To address these knowledge gaps, this study prepared biochars from rice husk, corn straw and corn cob to construct three BBCs with *P. chlororaphis* for triclocarban degradation. Multiscale analyses, including structural (SEM, BET), interfacial (FTIR, XPS), electrochemical (CV, EIS) and metabolic (LC-MS) characterizations, were employed to examine how biochar properties regulate the microstructure, surface chemistry and cellular metabolism. Specifically, this work aims to (i) compare the physicochemical characteristics and interfacial properties of RH-, CS- and CC-derived biochars; (ii) elucidate how these distinct carriers modulate EET and metabolic pathways in *P. chlororaphis* during TCC removal using electrochemical analysis and non-targeted metabolomics; and (iii) establish a feedstock-dependent structure–interface–metabolism coupling framework that links electron generation, transfer and regeneration with the pollutant removal performance. From an environmental chemical engineering perspective, this multiscale framework is expected to clarify how biochar regulates EET, metabolic reconfiguration and pollutant transformation. Such understanding is essential for rationally tailoring biochar materials and pyrolysis conditions, and for designing biochar-supported biofilm reactors and bioelectrochemical treatment units for cleaner production and cost-effective remediation of wastewater and groundwater contaminated with persistent micropollutants such as TCC.

## 2. Results

### 2.1. Physicochemical Properties of Biochar

The physicochemical properties of CS, CC, and RH biochars are shown in Table 1.

Distinct differences in morphology and surface chemistry were observed among the three biochars. On a mass basis including ash, CS and CC were carbon-rich (83.00–83.44 wt%), with relatively low oxygen contents (9.95–11.93 wt%), while RH exhibited a much lower carbon fraction (C ≈ 53.90 wt%) together with higher oxygen (O ≈ 33.30 wt%) and ash (10.67 wt%). The atomic H/C ratios were 0.148 (CS), 0.148 (CC), and 0.269 (RH), indicating a higher degree of carbonization for CS and CC than RH. The atomic O/C ratios were 0.090 (CS), 0.107 (CC), and 0.463 (RH), and the polarity index (O + N)/C followed the same order (0.104, 0.120, and 0.549, respectively). Notably, the difference-based oxygen content may include contributions from mineral phases in RH; therefore, O-related indices for RH should be interpreted with caution.

**Figure 1:**
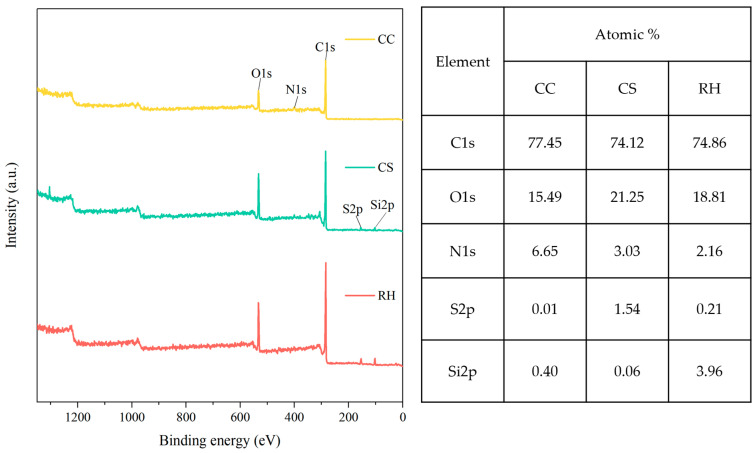
XPS survey spectra of the pristine biochars derived from corn cob (CC), corn straw (CS), and rice husk (RH). Major elemental peaks (C 1s, O 1s, N 1s, S 2p) and mineral-associated signals (Si 2p) are labeled.

XPS survey spectra and surface elemental quantification (Figure 1) showed clear feedstock-dependent differences in near-surface composition. All biochars were dominated by C and O, consistent with carbonaceous matrices bearing oxygenated surface species. CS exhibited the highest surface O fraction, CC showed the highest surface N fraction, and RH displayed a pronounced Si signal, consistent with silica/ash exposure typical of rice-husk-derived chars (C 1s 74.12–77.45 at%; O 1s 15.49–21.25 at%; N 1s 2.16–6.65 at%; Si 2p up to 3.96 at%). Minor heteroatom signals (e.g., S) also differed among feedstocks. These XPS results provide a surface-sensitive complement to FTIR and are used for cross-sample comparison of near-surface elemental signatures in later discussion.

Among them, CS displayed the largest specific surface area (85.51 m^2^/g), pore volume (3.8 cm^3^/g), and average pore diameter (139.68 nm), and the highest pH (9.46), followed by RH and then CC. Overall, although RH and CC had a lower surface area and porosity, their surface chemistry may still influence adsorption; however, the adsorption performance should be interpreted jointly with the pore structure and surface functionality. The BET values measured in this work fall within the ranges reported for comparable biochars in the literature [26,27,28].

**Figure 2:**
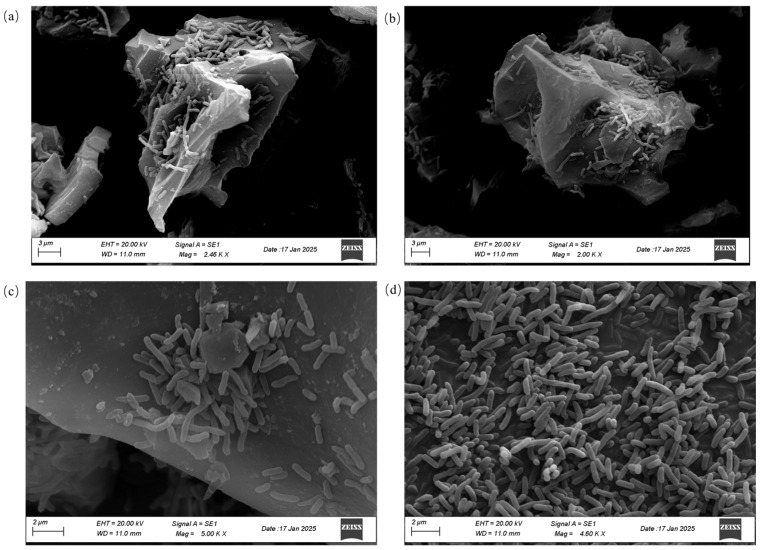
SEM of *P. chlororaphis* cells attached to different biochar carriers. (**a**) CS + PC composite, (**b**) CC + PC composite, (**c**) RH + PC composite, and (**d**) free PC (without biochar). The micrographs shown are representative of multiple randomly selected regions from independently prepared samples.

Figure 2 presents SEM images of PC, CS + PC, CC + PC, and RH + PC. All three biochars exhibited well-developed porous structures that provided suitable microenvironments for bacterial adhesion and biofilm formation, though their surface morphologies varied considerably.

In the CS + PC sample (Figure 2a), abundant surface grooves and pores were observed, with cells aggregated along layered edges, forming moderately dense clusters. The CC + PC sample (Figure 2b) displayed stratified flakes with abundant pores/voids. Pore size distribution analysis indicated that CC was dominated by mesopores (2–50 nm) with a negligible micropore contribution (<2 nm), consistent with its relatively low BET surface area (12.96 m^2^/g) but measurable total pore volume (0.6 cm^3^/g) and average pore diameter of 14.96 nm. Within these pores/voids, cells appeared to form more continuous biofilm-like layers in representative SEM fields, suggesting that CC may provide a favorable surface for colonization. In contrast, the RH + PC sample (Figure 2c) exhibited a smoother surface with smaller pores, and the attached cells were more sparsely distributed. The control group (PC, Figure 2d) showed dispersed individual cells in the absence of carrier support or biofilm formation.

Notably, the comparisons of cell attachment/biofilm coverage among carriers are based on representative SEM images and are therefore qualitative; rigorous quantification of surface coverage (e.g., ImageJ, version 1.54 based area fraction analysis across multiple fields of view and replicates and/or complementary biomass assays) will be included in future work.

Overall, the pore structure of the biochar strongly influenced bacterial attachment and spatial distribution. Among the three composites, the highly porous network and greater surface roughness of CC biochar appeared to promote denser biofilm-like coverage in representative SEM fields, thereby supporting subsequent interfacial electron exchange. Such structure–function relationships provide a basis for selecting or blending biochars tailored to specific reactor configurations.

### 2.2. TCC Removal Performance

**Figure 3:**
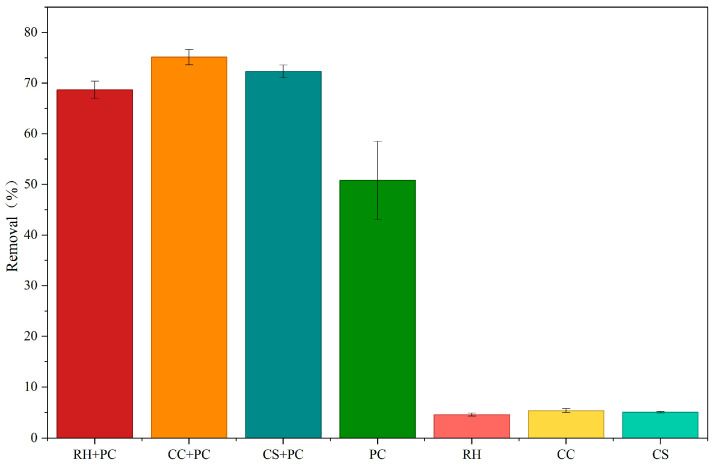
TCC removal by different BBCs and corresponding biochars. Bar plots show TCC removal (%) after 48 h for RH + PC, CC + PC, CS + PC, PC (cells only), and abiotic controls (RH, CC and CS). For biotic treatments, the observed removal reflects biodegradation together with a small apparent (non-degradative) loss, as indicated by the abiotic controls. Data represent the mean of four parallel replicate cultures (*n* = 4), and error bars indicate standard deviation (SD). Compared with PC, all BBCs exhibited markedly higher TCC removal, demonstrating that biochar loading effectively enhanced the degradation activity of *P. chlororaphis*. The composite systems showed comparable performances with minor differences and good stability, following the overall trend CC + PC > CS + PC > RH + PC > PC. In contrast, systems containing biochar alone exhibited low TCC removal, indicating that biochar itself contributed little to TCC degradation, and that the enhancement primarily resulted from synergistic interactions between the biochar and the bacterial cells.

The TCC removal performance of the BBCs is shown in Figure 3.

In this study, “TCC removal (%)” was defined as the decrease in extractable parent TCC quantified by whole-slurry extraction followed by HPLC (aqueous + sorbed extracted together). Abiotic biochar-only controls showed only 4.62–5.40% apparent loss after 48 h, which is attributed mainly to method recovery/handling loss rather than true degradation. Therefore, removal in biotic treatments is discussed primarily as parent-TCC biotransformation while acknowledging a minor non-degradative component at the level indicated by the abiotic controls; adsorption was not separated or subtracted, and transformation products were not quantified.

The observed performance trend was reproducible across independent replicate cultures and independently prepared composites under the tested batch conditions.

### 2.3. Functional Group Changes Observed in FTIR Spectra

The surface functional groups of the BBCs play an important role in electron transfer and environmental reactivity. FTIR comparisons between the biochars and their composites indicated clear changes in hydroxyl (O-H), amino (N-H), carboxyl (-COOH), amide (-CONH-), carbonyl (C=O), and aromatic C=C groups. These changes in O- and N- containing groups may alter the abundance and accessibility of interfacial functionalities, which could contribute to improved interfacial electron-transfer kinetics when considered together with the electrochemical results.

**Figure 4:**
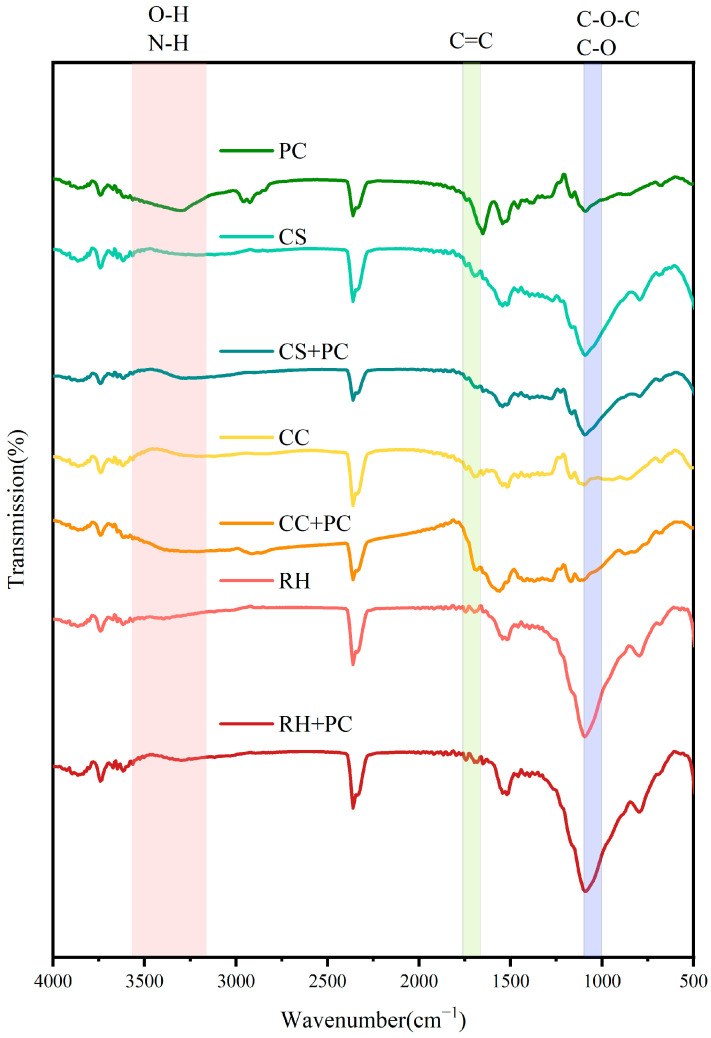
FTIR spectra of PC (cells only), biochars, and BBCs. Spectra are shown for PC (cells only), CS, CS + PC, CC, CC + PC, RH and RH + PC. Main absorption bands appear at ~3400 cm^−1^ (O-H/N-H), ~1600 cm^−1^ (aromatic C=C/amide), and 1000–1100 cm^−1^ (C-O/C-O-C). Spectra are representative of independently prepared samples showing reproducible spectral features.

As shown in Figure 4, the FTIR spectra of PC, CS, CC, RH, and their respective composites displayed typical absorption bands within 500–4000 cm^−1^, but distinct differences in peak shape and intensity were observed. These variations reflect alterations in the surface chemical environment caused by the interaction between biochar carriers and microbial cells.

Within each biochar type, all composite systems exhibited reductions or shifts in the absorption bands of hydroxyl, carbonyl, and carboxyl groups compared with their corresponding pure biochars and bacterial cells, suggesting that cell immobilization considerably affected the surface chemical activity of the carriers. In particular, the broad peak around 3420 cm^−1^ (O-H/N-H stretching) markedly weakened in all composites. Bands at 1690 cm^−1^ (C=O and aromatic C=C vibrations) decreased in intensity and showed minor blue shifts, while the signal at 1384 cm^−1^ (carboxyl and amide vibrations) attenuated only in the composites. In the 1000–1100 cm^−1^ region (C-O-C/C-O vibrations from polysaccharides and nucleic acids), peak broadening and slight position shifts were also evident.

Comparisons among different BBCs demonstrated distinct interfacial functional-group responses. The CC + PC system, with its highly aromatic carbon skeleton, exhibited the most prominent spectral changes. Although the RH + PC system was less aromatic and lower in carbon content, its abundant polar oxygenated groups and siliceous components also contributed to notable functional-group variations.

The apparent increase in signals associated with oxygen-containing functionalities (e.g., C=O and C-O related bands) suggests more polar interfacial chemistry after cell immobilization. Based on prior studies, quinone-/phenolic-like moieties on carbonaceous materials have been reported to participate in reversible redox cycling and to serve as redox mediators (electron shuttles) in EET-related processes [29,30,31]. However, FTIR alone cannot uniquely identify quinone structures or quantify specific redox-active sites; therefore, the present FTIR results are discussed as qualitative evidence of interfacial functional-group involvement, and the proposed redox-mediation mechanism is supported primarily by the literature together with the electrochemical trends.

Overall, FTIR indicates that all BBCs altered interfacial functional groups upon immobilization, which may contribute to improved interfacial electron transfer when considered together with the electrochemical results.

### 2.4. Comparative CV and EIS Characteristics

Electrochemical analyses confirmed that all BBCs significantly enhanced electron transfer capability compared with the pure bacterial culture.

As shown in Table 2, the solution resistance (Rs) of all samples ranged from 30.7 to 41.2 Ω, indicating a consistent electrolyte environment across the systems. In contrast, the charge transfer resistance (Rct) varied markedly. The PC group displayed the highest Rct (254.0 kΩ), reflecting unfavorable interfacial charge-transfer kinetics and thus sluggish electron transfer under the tested conditions. The Rct values of all biochar-loaded systems were substantially lower, with RH + PC exhibiting the lowest Rct (5.6 kΩ)—much lower than those of pure RH (51.2 kΩ) and PC, suggesting that biochar loading effectively facilitated interfacial electron transfer (reduced Rct).

**Figure 5:**
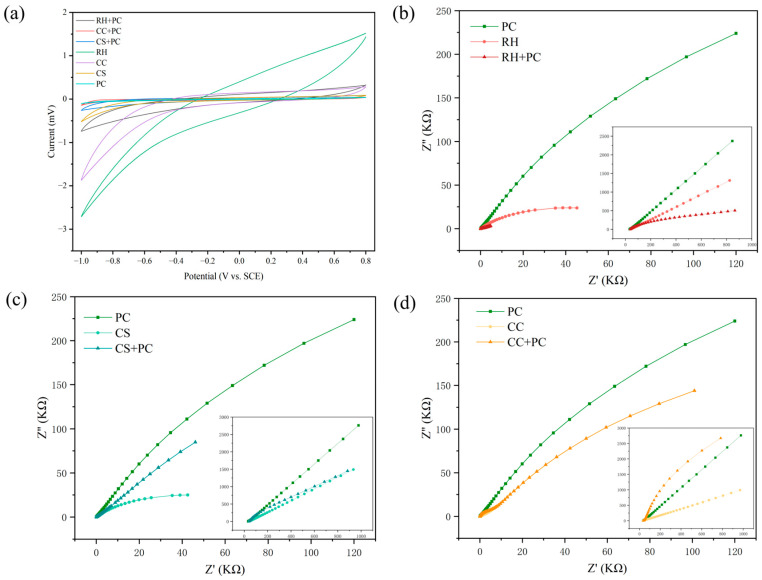
Electrochemical behavior of *P. chlororaphis*, biochars, and biochar–bacteria composites. (**a**) CV of PC, CS, CC, RH and their composites in 0.1 mol/L KCl at a scan rate of 50 mV/s. (**b**–**d**) EIS Nyquist plots comparing PC with each biochar and its composite: (**b**) PC, CC and CC + PC; (**c**) PC, CS and CS + PC; (**d**) PC, RH and RH + PC. All curves are representative of reproducible measurements from independently prepared electrodes.

As shown in Figure 5a, the CV of PC exhibited negligible current near the baseline, indicating limited interfacial electron exchange. In contrast, all pure biochars presented distinct redox peaks, reflecting their inherent electrochemical activity. The composite systems displayed intermediate current responses, positioned between those of the pure bacterial cells and biochars, implying that biochar loading and immobilization improved electrochemically active interfacial contact, thereby facilitating EET [32].

The EIS results further supported this trend. As illustrated in Figure 5b–d, all Nyquist plots presented characteristic semicircles followed by linear tails, representing concurrent charge-transfer and ion-diffusion processes [33]. The composite systems showed notably smaller semicircles than PC, confirming a marked decrease in Rct. Among them, RH + PC exhibited the smallest semicircle and the steepest slope in the low-frequency region, consistent with its lowest Rct and steeper low-frequency tail, suggesting faster interfacial charge-transfer kinetics and distinct mass-transport behavior under the tested conditions. Conversely, CC + PC presented a slightly higher Warburg impedance (Zw), suggesting weaker pore connectivity, whereas RH + PC and CS + PC demonstrated lower Zw values, indicating superior ionic and interfacial diffusion properties.

The Rct of the RH + PC system was markedly lower than those reported of most biochar-based conductive materials in the literature, which are typically in the range of several thousand to tens of thousands of ohms, highlighting a substantial improvement in interfacial charge-transfer efficiency [34]. This superior performance can be attributed to the electron–ion coupling interface formed synergistically between the porous carbon framework and the cell surface functional groups [35]. The porous carbon framework of RH biochar may improve electrochemically active contact and electrolyte access, while surface oxygenated groups may facilitate interfacial coupling with cell-surface components, collectively contributing to a lower fitted Rct. Together, these effects enhanced interfacial electron and ion exchange, thereby greatly reducing Rct. Similar studies have also shown that the integration of biochar with other carbonaceous materials improves electrochemical performance by optimizing conductive networks and interfacial contact [36,37].

Interestingly, although RH + PC exhibited the lowest Rct, its TCC degradation rate was lower than those of CC + PC and CS + PC. This discrepancy between conductivity and degradation performance indicates that improved bulk conductivity alone does not guarantee enhanced pollutant removal. Two additional factors may account for this behavior: (i) Notably, the ranking of fitted Rct did not fully mirror the ranking of parent-TCC removal, indicating that interfacial charge-transfer kinetics are an enabling factor but not the sole determinant of removal performance. (ii) In contrast, CC- and CS-derived biochars possess more abundant quinone- and phenolic-like groups that can act as redox mediators, thereby facilitating EET and strengthening the coupling between electron flow and TCC transformation [38,39].

In summary, the CC + PC system achieved the most balanced configuration between electrical conductivity, interfacial chemistry, and microenvironmental compatibility, representing an optimal synergy between electron transfer efficiency and biodegradation activity. Therefore, the interplay among the biochar’s electrochemical properties, interfacial structure, and microbial activity is the key determinant of overall degradation performance. This inference was further supported by the subsequent metabolomic analysis.

### 2.5. Global Metabolomic Alterations Identified by Untargeted Metabolomics

**Figure 6:**
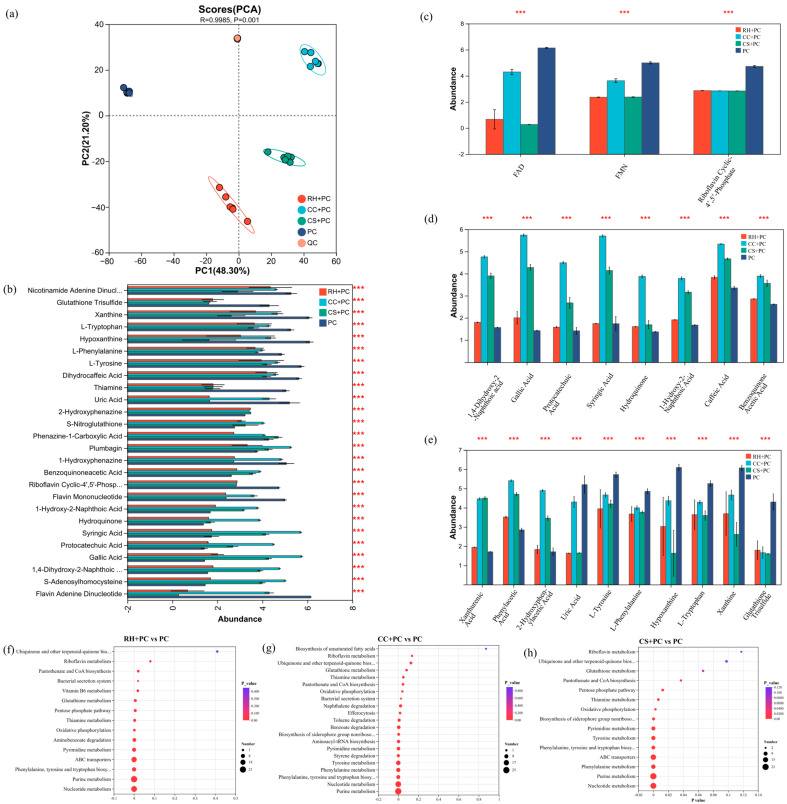
Metabolomic profiling and KEGG pathway enrichment of biochar–bacteria composites (BBCs) compared with pure *P. chlororaphis*. (**a**) PCA score plot showing separation of RH + PC, CC + PC, CS + PC, PC and QC samples based on untargeted LC-MS data (*n* = 6 per group). (**b**) Differential metabolites between biochars (RH, CC, CS) and PC. (**c**) Changes in flavin-derived electron-shuttle metabolites. (**d**) Changes in quinone- and polyphenol-associated metabolites. (**e**) Differential metabolites related to aromatic amino acids, purines, urea and glutathione. (**f**–**h**) KEGG pathway enrichment of differential metabolites between BBCs and PC: (**f**) RH + PC vs. PC, (**g**) CC + PC vs. PC, and (**h**) CS + PC vs. PC. Differential metabolites were selected using VIP > 1 and *p* < 0.05. *** indicates statistical significance at *p* < 0.001.

Untargeted LC-MS analysis revealed substantial remodeling of the extracellular metabolome in *P. chlororaphis* following immobilization on biochar carriers [40]. In the unsupervised principal component analysis (PCA) (Figure 6a), QC samples clustered tightly, whereas the biochar-loaded and free-cell groups separated clearly, indicating that biochar profoundly reshaped the extracellular metabolic profile of the cells.

As shown in Figure 6b–e, relative to PC, most differential metabolites in the three composite systems were associated with three major functional themes: (i) restructuring of soluble electron shuttles; (ii) expansion of aromatic quinone/polyphenol-associated transformation products; and (iii) coordinated regulation of metabolites related to cofactor and antioxidant homeostasis [41]. Because this is an untargeted metabolomics dataset, the results primarily reflect relative abundance changes rather than direct metabolic flux.

At the level of flavin-based electron shuttles, the relative abundances of FAD and FMN generally declined. The decrease was minimal in the CC system, suggesting that flavin-associated electron shuttling was comparatively maintained. The CS system exhibited a moderate reduction, consistent with a potential balance among flavins, siderophores, and ABC transport. The RH system showed the largest decline, suggesting a stronger redistribution of metabolic resources under this condition.

In contrast, quinone- and polyphenol-associated metabolite features increased markedly in all BBCs. Based on differential metabolite patterns and KEGG enrichment, the prominence of quinone/polyphenol-associated signatures followed the order CC + PC > CS + PC > RH + PC.

**Figure 7:**
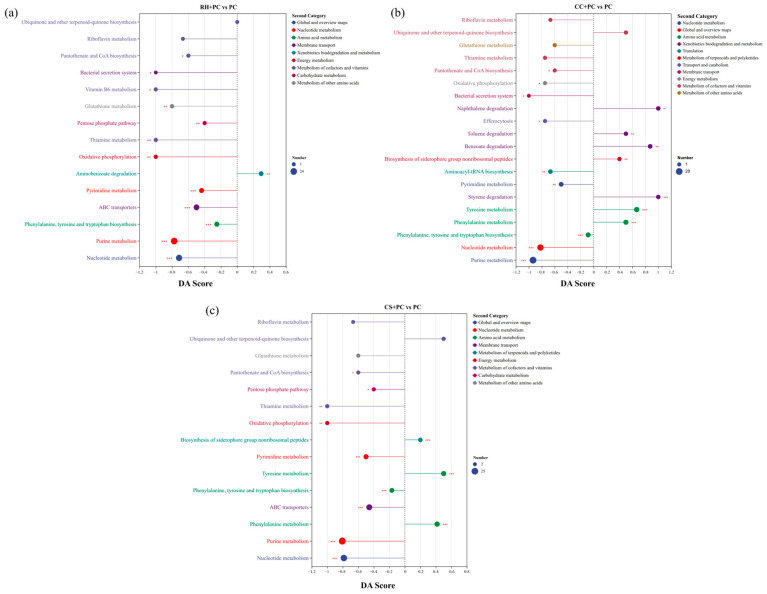
KEGG pathway activity trends derived from DAscore analysis for BBCs relative to pure *P. chlororaphis*. (**a**) RH + PC vs. PC, (**b**) CC + PC vs. PC, and (**c**) CS + PC vs. PC. Positive DAscores indicate relative pathway activation and negative values indicate relative suppression in BBCs compared with PC. Dot color denotes KEGG pathway category, and dot size represents the number of contributing differential metabolites. Asterisks indicate statistical significance: * *p* < 0.05, ** *p* < 0.01, and *** *p* < 0.001.

In the RH + PC system (Figure 6f and Figure 7a), the pathway-level patterns were consistent with enhanced redox/antioxidant maintenance. Enrichment of aromatic-cleavage pathways was weak, with only the aminobenzoate degradation (map00627, P_adjust_ = 0.0107, DA Score = +0.29) significantly upregulated, suggesting limited aromatic oxidation activity. In contrast, the pentose phosphate pathway (PPP, map00030, P_adjust_ = 0.0288, DA Score = −0.40) and glutathione metabolism (GSH, map00480, P_adjust_ = 0.0298, DA Score = −0.80) showed significantly negative DA Scores. These results are consistent with a shift in the relative metabolic state toward re-dox-homeostasis-associated processes; however, they do not quantify NADPH/GSH cycling rates. Oxidative phosphorylation (map00190, P_adjust_ = 0.0099, DA Score = −1) also displayed a significant negative DA Score, indicating an altered energy-metabolism signature in RH + PC compared with PC. Overall, the RH + PC system exhibited a PPP-GSH-associated metabolic signature consistent with increased redox demand and antioxidant maintenance [42].

The CC + PC system (Figure 6g and Figure 7b) accumulated the largest quantities of naphthoquinones, benzoquinones, and polyphenol oxidation products (e.g., 1,4-dihydroxy-2-naphthoic acid, benzoquinone acetic acid, gallic acid, and protocatechuic acid). Pathways related to aromatic cleavage and quinone formation were among the most positively directed, including aminobenzoate (map00627, P_adjust_ = 6.1 × 10^−6^, DA Score = +0.65), styrene (map00643, P_adjust_ = 0.0033, DA Score = +1), benzoate (map00362, P_adjust_ = 0.022, DA Score = +0.88), and naphthalene degradation (map00626, DA Score = +1). Phenolic compound and aromatic amino acid metabolism (map00350, 00360) also showed positive DA Scores, supporting a metabolic profile consistent with enhanced formation/accumulation of redox-active aromatic intermediates and mediators [43]. Although PPP (map00030, DA Score = −0.6) and glutathione metabolism (map00480, DA Score = −0.6) were not significantly enriched, their negative DA Scores are suggestive of altered redox-related metabolite patterns rather than direct evidence of continuous consumption or increased flux.

The CS + PC system (Figure 6h and Figure 7c) exhibited coordination changes in pathways associated with electron-donor generation and export, with moderate aromatic-degradation signatures. Polyphenols such as gallic, caffeic, and syringic acids increased significantly. Multiple aromatic-compound degradation pathways were enriched, including styrene (map00643, P_adjust_ = 0.0020, DA Score = +1) and aminobenzoate degradation (map00627, P_adjust_ = 1.36 × 10^−5^, DA Score = +0.59). Phenylalanine and tyrosine metabolism (map00360, 00350) were also positively directed, consistent with aromatic oxidation/quinonization. Enrichment of siderophore biosynthesis (map01053, P_adjust_ = 0.0020, DA Score = +0.2) and ABC transporters (map02010, P_adjust_ = 6.6 × 10^−7^, DA Score = −0.46) suggested reinforced iron–polyphenol-assisted interactions and transmembrane transport, which may support sustained extracellular redox mediation. Although PPP (map00030, P_adjust_ = 0.053, DA Score = −0.4) and glutathione metabolism (map00480, P_adjust_ = 0.17, DA Score = −0.6) were negatively directed, these patterns are best interpreted as relative changes in metabolite abundance associated with redox balance.

Linking these metabolic patterns with pollutant removal performance, the three composites showed distinct profiles. The CC + PC system displayed the strongest signatures of aromatic degradation/quinone formation and the highest abundance of redox-active aromatic intermediates, which may contribute to improving extracellular redox mediation and corresponded to the highest TCC removal. The CS + PC system showed moderate aromatic-degradation signatures together with siderophore/ABC-transporter enrichment, which may support sustained electron export and stable TCC removal. The RH + PC system exhibited stronger PPP-GSH associated redox-maintenance signatures but weaker aromatic-degradation signatures, corresponding to slightly lower TCC removal. Overall, the metabolomics results suggest that biochar immobilization is associated with a reorganization of extracellular redox-related metabolites, which likely contributes to differences in electron transfer potential and pollutant removal among the BBCs. Previous studies have shown that biochar can influence the extracellular electron transfer and redox balance in bio-electrochemical and related systems [44].

Integration of the metabolic and removal data suggests that although all three BBCs exhibited adaptive shifts in energy/redox-related metabolism, they adopted distinct strategies. CC + PC exhibited the most prominent aromatic/quinone-associated mediator signature, CS + PC presented a more balanced mediator/transport-associated signature, and RH + PC showed a stronger PPP/GSH-associated redox-maintenance signature. This gradient aligned with the observed TCC degradation performance, providing mechanistic insight into system-level differences.

## 3. Discussion

In summary, structural and chemical differences among biochars derived from distinct feedstocks regulated the behavior of immobilized *P. chlororaphis* systems and was associated with distinct electron-transfer-related interfacial and metabolic signatures that can be interpreted within an electron generation–transfer-regeneration framework. As a result, a functionally diversified and synergistic EET network was established. Each system exhibited a distinct emphasis: the CC-based system was characterized by aromatic cleavage and quinonization, showing the strongest electron generation; the CS-based system achieved relatively stable output through iron–polyphenol complexation and transmembrane ABC transport; and the RH-based system featured rapid regeneration via the PPP-GSH cycle.

Regarding TCC degradation, the three BBCs displayed a clear performance gradient. All biochar-loaded systems markedly improved TCC degradation compared with the free-cell system, following the order CC + PC > CS + PC > RH + PC.

Based on the combined structural, spectroscopic, electrochemical, and metabolomic results, we propose an interpretive four-layer “structure–interface–metabolism” coupling framework to rationalize the feedstock-dependent differences in TCC removal. This framework is intended to organize the observations across scales rather than to claim a fully resolved causal mechanism, and each layer is anchored to measured descriptors as follows.

i. Structural confinement framework:

This layer is supported by the BET/porosity metrics and the SEM attachment patterns. For example, CS exhibited the largest BET surface area (85.51 m^2^/g) and pore volume (3.8 cm^3^/g), whereas CC showed a lower BET area (12.96 m^2^/g) but mesopore-dominated structure (2–50 nm) with a measurable pore volume (0.6 cm^3^/g) and an average pore diameter of 14.96 nm. Correspondingly, SEM images indicated feedstock-dependent differences in cell spatial distribution: CS + PC showed aggregation along grooves/edges, CC + PC exhibited more continuous biofilm-like layers within pores/voids in representative fields, and RH + PC showed comparatively sparse attachment.

ii. Interfacial synergy layer:

This layer is supported by reproducible interfacial chemical changes observed after immobilization in FTIR spectra (Figure 4), including attenuation of the broad O-H/N-H stretching band (~3420 cm^−1^), decreased intensity with minor shifts near ~1690 cm^−1^ (C=O/aromatic C=C), attenuation at 1384 cm^−1^ (carboxyl/amide), and broadening/shifts in the 1000–1100 cm^−1^ region (C-O/C-O-C). Together, these spectral features indicate that immobilization alters the interfacial chemical environment (e.g., hydrogen bonding/complexation and EPS-related functionalities). Consistent with prior studies, quinone-/phenolic-like moieties on carbonaceous materials may participate in reversible redox cycling and thereby contribute to mediated/interfacial electron transfer; however, FTIR alone cannot uniquely identify quinone structures, so redox mediation is treated here as a plausible contribution rather than a direct FTIR-derived conclusion [45]. Notably, electrochemical interfacial kinetics (e.g., fitted Rct) did not fully mirror the trend in parent-TCC removal: although RH + PC showed the lowest fitted Rct, its parent-TCC removal was not the highest. This indicates that Rct is an enabling descriptor but not a sufficient predictor of removal performance. Feedstock-dependent surface chemistry and mineral exposure (e.g., the stronger Si signal for RH) may influence the accessibility of carbon-surface redox sites and/or the quality of cell–carrier contact, thereby modulating how interfacial electron exchange couples with pollutant transformation. This interpretation remains hypothesis-level and requires targeted validation.

iii. Metabolic regulation layer:

This layer is supported by the untargeted metabolomics statistics: tight QC clustering and clear group separation in PCA (Figure 6a; *n* = 6 per group), differential metabolites selected using VIP > 1 and *p* < 0.05 (Figure 6b–e), and pathway-level enrichment/directionality (Figure 6f–h and Figure 7) with adjusted *p* values and DA Scores. For instance, CC + PC showed the strongest enrichment and positive directionality for aromatic degradation/quinone-associated pathways (e.g., aminobenzoate degradation with P_adjust_ = 6.1 × 10^−6^, DA Score = +0.65; styrene degradation P_adjust_ = 0.0033, DA Score = +1), whereas RH + PC showed patterns more consistent with redox/antioxidant maintenance signatures. Because the dataset is untargeted, these results indicate relative abundance changes rather than direct fluxes.

iv. Energy integration layer:

This layer is supported by the electrochemical descriptors (Table 2; Figure 5). Relative to PC (Rct = 2.54 × 10^5^ Ω), all BBCs substantially reduced interfacial charge-transfer resistance. RH + PC exhibited the lowest Rct (5.64 × 10^3^ Ω), followed by CS + PC (9.65 × 10^4^ Ω) and CC + PC (1.76 × 10^5^ Ω), indicating improved interfacial electron-transfer kinetics upon biochar loading, while Nyquist low-frequency tails and fitted Zw reflect differences in ion diffusion/mass transport. Importantly, we interpret Rct as an interfacial charge-transfer parameter, not as bulk conductivity. Together with CV redox features of the pure biochars and composites (Figure 5a), these results support that the biochar–cell interface facilitates electron exchange, but they do not uniquely resolve DET versus mediated pathways.

In summary, the four-layer framework is a structured interpretation that integrates (i) measured pore/attachment descriptors, (ii) FTIR-observed interfacial chemical changes, (iii) statistically supported metabolomic shifts, and (iv) electrochemical kinetics descriptors. It provides a coherent explanation for the observed performance trend (CC + PC > CS + PC > RH + PC > PC) while remaining open to future validation using targeted mediator quantification and methods that can more directly discriminate electron-transfer pathways.

**Figure 8:**
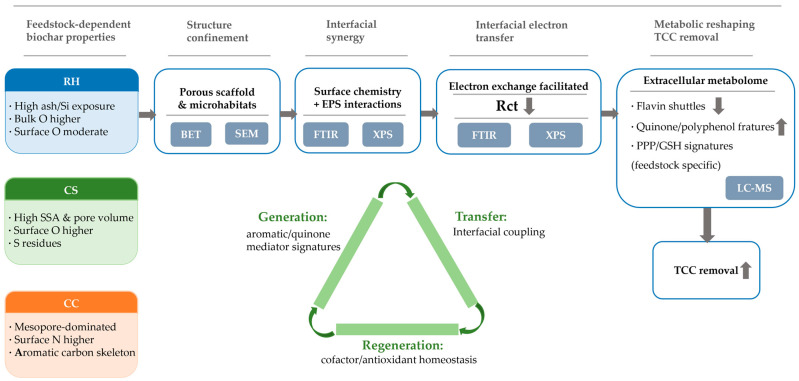
Evidence-supported mechanistic pathway for feedstock-dependent enhancement of triclocarban (TCC) removal by biochar–*Pseudomonas chlororaphis* composites. Upward arrows indicate an increase or enhancement, downward arrows indicate a decrease or reduction.

To improve mechanistic clarity, Figure 8 summarizes an evidence-supported pathway by which feedstock-derived biochar properties modulate extracellular electron transfer (EET) and redox-related metabolic signatures in the immobilized *P. chlororaphis* systems, thereby enhancing parent-TCC removal. Briefly, biochar provides (i) a porous scaffold that concentrates cells and shortens diffusion distances (BET/SEM), and (ii) a chemically active interface where oxygen-/nitrogen-containing surface signatures and mineral exposure (FTIR/XPS) promote biomolecule interactions and interfacial coupling. These structural/interfacial features are reflected in improved electrochemical descriptors (CV redox features and decreased fitted Rct in EIS), indicating facilitated interfacial electron exchange. Concurrently, untargeted extracellular metabolomics indicate a redistribution of redox-associated metabolite signatures, characterized by reduced reliance on soluble flavin shuttles and increased quinone/polyphenol-associated features, together with pathway-level patterns suggestive of distinct redox-maintenance strategies among feedstocks. Within this integrative interpretation, “electron generation” corresponds to enhanced formation/availability of redox-active aromatic/quinone-like mediator signatures, “electron transfer” corresponds to strengthened biochar–cell interfacial coupling (lower Rct), and “electron regeneration” corresponds to cofactor/antioxidant-homeostasis-associated signatures (e.g., PPP/GSH-related patterns). We stress that this pathway is intended to organize the multi-scale observations rather than to uniquely prove causality; discriminating DET versus mediated pathways and quantifying intracellular redox couples (e.g., NAD(P)H/NAD(P)^+^, GSH/GSSG) will be pursued in future targeted studies.

These interpretations are aligned with the established literature showing that biochar can enhance microbial EET not only by providing a conductive scaffold but also through redox-active surface functionalities (e.g., quinone-/phenolic-like moieties) that can participate in reversible redox cycling and facilitate mediated or interfacial electron exchange [7,30,36]. In addition, shifts involving the pentose phosphate pathway and glutathione metabolism are widely linked to cellular redox homeostasis via NADPH supply and antioxidant buffering [42]. Therefore, we frame the “electron generation/transfer/regeneration” description as an organizational interpretation consistent with both the literature and our multi-omics/electrochemical trends.

Compared with previous studies that enhanced EET mainly through conductive materials such as carbon felt or carbon paper [46,47], this work suggests that biochar may function not only as a conductive scaffold but also as a modulator of the microbial metabolic state. Based on the combined trends observed in CV/EIS responses and extracellular metabolite profiles, we propose an interpretive framework in which all BBCs may involve contributions from direct electron transfer (DET), interfacial mediated electron transfer (ICET), and metabolically associated redox processes (MET). However, we acknowledge that the current electrochemical and untargeted metabolomics data do not uniquely resolve or directly discriminate among these mechanisms. Within this framework, CC-based systems showed patterns consistent with a relatively greater contribution of quinone-/aromatic-intermediate-associated redox mediation, whereas CS-based systems exhibited more prominent interfacial/mediator-related features suggestive of ICET contributions, and RH-based systems displayed more pronounced PPP/GSH-associated redox-maintenance signatures, which may be associated with MET-like behavior. These tendencies are not mutually exclusive and should be interpreted as relative contributions rather than definitive assignments. Collectively, the BBCs can be viewed as a coupled structure–interface–metabolism system, where biochar provides electron-conducting/transport pathways and may influence redox-related metabolic signatures, offering guidance for designing tunable biochar-enabled bioelectrochemical remediation platforms.

To avoid overinterpretation, we distinguish here between observations directly supported by our measurements and hypothesis-level mechanistic interpretations. This work demonstrates (i) enhanced TCC removal by biochar–bacteria composites relative to free cells (Figure 3), (ii) substantially reduced interfacial charge-transfer resistance and altered electrochemical responses upon immobilization (Table 2; Figure 5), (iii) reproducible FTIR band changes indicating interfacial chemical perturbations (Figure 4), and (iv) statistically supported extracellular metabolomic remodeling (PCA separation; VIP > 1 and *p* < 0.05; KEGG enrichment with P_adjust_ and DA-score directionality; Figure 6 and Figure 7). In contrast, attributing “enhanced electron generation” specifically to quinonization/quinone-like moieties and attributing “improved regeneration” specifically to PPP–GSH activation remain interpretive hypotheses consistent with these trends, because quinone-like site density and intracellular redox couples (e.g., NAD(P)H/NAD(P)^+^ and GSH/GSSG) were not directly quantified in this study.

Previous studies have reported TCC removal via a range of approaches, including adsorption-oriented carbon materials [48,49], advanced oxidation/photocatalytic processes [49,50], and biological treatment using activated sludge or specialized degraders [49,51]. Compared with these reports, the present work does not aim to claim the highest absolute removal under all conditions; rather, it contributes a mechanistically organized, feedstock-dependent understanding of how biochar carriers can couple microstructure, interfacial chemistry, electrochemical behavior, and extracellular metabolomic signatures to improve TCC removal by an immobilized bacterial system. Importantly, because reported performances depend strongly on the water matrix, initial concentration, biomass loading, contact time, and whether co-substrates are supplied, cross-study numerical comparisons should be interpreted cautiously. Within these constraints, our results indicate that agricultural-residue-derived biochars can serve as low-cost supports that enhance interfacial electron-transfer descriptors (e.g., reduced fitted Rct) and reshape redox-related metabolic signatures, thereby improving parent-TCC removal in batch microcosms.

Despite these advances, several limitations remain. First, leachable dissolved organic carbon (DOC) from the biochars was not quantified. Although the biochars were repeatedly washed until the wash-water conductivity stabilized, this does not rule out residual leachable organics, which have been reported to range from low mg/L to tens of mg/L depending on the feedstock, pyrolysis conditions and aging; thus, a DOC contribution of a magnitude comparable to (or lower than) the background carbon available to the cells cannot be excluded [52]. Such leached DOC could (i) provide an auxiliary carbon/energy source [53], (ii) introduce redox-active aromatics/phenolics that act as extracellular mediators [38], and/or (iii) change the solution chemistry (pH/complexation) and thereby indirectly affect degradation [54]. Consequently, part of the observed metabolomic remodeling and performance enhancement may include an overlay of “biochar-leachate effects” in addition to immobilization/interface effects. To better constrain this factor, future work will quantify DOC/TOC and include leachate-only and pre-extracted/aged-biochar controls to decouple leached effects from solid-surface/interface effects. Second, the metabolomic results are based on relative abundances and do not quantify key redox intermediates or intracellular couples (e.g., quinones/polyphenols, GSH/GSSG, and NADPH/NADP^+^). Targeted quantification and perturbation/flux-oriented assays are needed to validate the proposed quinonization- and PPP-GSH-related interpretations. In addition, potential contributions of heteroatoms/trace metals to interfacial electron exchange were not isolated and warrant further validation, and future work will include HR-XPS of composites and AFM/conductive-AFM mapping to resolve nanoscale adhesion and local conductivity heterogeneity [55]. Third, transformation products of TCC and the mass balance of TCC were not characterized. Therefore, although the decrease in extractable parent TCC indicates that improved removal, dominant pathways, intermediate yields/accumulation, and dichlorination could not be resolved. Future work will conduct time-resolved product identification/quantification (e.g., suspect/non-target screening followed by targeted LC–MS/MS) together with complementary indicators such as chloride release and, where relevant, bulk organochlorine metrics (e.g., AOX/TOX) and TOC/COD changes in controlled media and real wastewater matrices. Fourth, mixture-toxicity changes were not evaluated; standardized bioassays will be included in future studies to assess risk reduction alongside parent-compound removal. Fifth, reusability and long-term stability of the composites were not tested. Multi-cycle operation will be assessed to quantify activity decay/biomass retention and to characterize post-use changes in morphology and surface chemistry (e.g., SEM, FTIR/XPS, BET/porosity).

Overall, the BBCs can be described as a coupled structural–interfacial–metabolism network. The observations are consistent with coordinated changes in interfacial electron-transfer descriptors and extracellular redox-related metabolic signatures, accompanied by improved TCC removal. The proposed multilayer coupling model suggests an important role of biochar in microbial systems and provides theoretical and practical insights for designing high-efficiency, adaptive, bioelectrochemical remediation materials. From a sustainability perspective, the use of abundant agricultural residues as precursors for functional biochar carriers can reduce waste disposal burdens and create value-added materials for wastewater treatment. Future work should also evaluate the long-term stability, regeneration, and life-cycle environmental impacts of such biochar-supported systems in pilot-scale reactors to fully assess their feasibility for large-scale deployment.

## 4. Materials and Methods

### 4.1. Preparation and Characterization of Biochar and Biochar–Bacteria Composites

Biochar feedstocks were sourced from an agricultural by-product supplier in Henan Province, China. The raw materials were rinsed with deionized water, air-dried, and ground. Pyrolysis was carried out in a tubular furnace (SX2-12-10A box resistance furnace, Huyueming, Tianjin, China) under N_2_ (flow rate: 0.5 L/min) by heating from room temperature to 500 °C at 10 °C/min and holding at 500 °C for 120 min; samples were then cooled naturally to room temperature under continuous N_2_ flow. The obtained biochars were ground and sieved to 100–200 mesh; the particle-size fraction used in subsequent experiments was approximately 75–150 μm. To remove loosely bound soluble salts, the biochars were washed with ultrapure water by centrifugation (8000 rpm, 5 min), and the washing procedure was repeated until the conductivity of the supernatant stabilized below 30 μS/cm. The washed biochars were freeze-dried for 12 h and stored in a desiccator at room temperature until use.

To minimize potential interference from leachable organics, biochars were repeatedly washed as described above; nevertheless, residual low-ionic-strength organics cannot be fully excluded and are discussed as a limitation.

To prepare BBCs, 0.2 g of sterilized biochar was added to 100 mL of M9 medium in a 250 mL Erlenmeyer flask and autoclaved at 121 °C for 30 min. After cooling, 2 mL of *P. chlororaphis* suspension (OD600 = 2.51, corresponding to approximately 1.14 × 10^9^ CFU/mL; wet biomass = 40 g/L) was inoculated and incubated at 30 °C with shaking (150 rpm) for 24 h to promote bacterial adhesion (Figure 2). The mixture was then allowed to stand for 10–20 min, and the supernatant was discarded. The remaining solids were rinsed twice with sterile phosphate-buffered saline (PBS, pH 7.4), freeze-dried, and stored at 4 °C. All operations were carried out aseptically.

Surface morphology was examined using a field-emission scanning electron microscope (SEM, Zeiss EVO MA15, Carl Zeiss, Oberkochen, Germany) operated under high-vacuum conditions. Samples were coated with a thin layer of gold and imaged at 5–10 kV. Elemental composition and chemical states were determined by X-ray photoelectron spectroscopy (XPS, Thermo Scientific K-Alpha^+^, Waltham, MA, USA) with an energy range of 5–1500 eV and an energy resolution of ≤0.5 eV. The C 1s peak at 284.8 eV was used for binding-energy calibration. XPS spectra were collected from an analysis area of approximately 55 μm in diameter. Bulk elemental composition (C, H and N; wt%) of the biochars was determined using an elemental analyzer (Vario MICRO cube, Elementar Analysensysteme GmbH, Langenselbold, Germany). Ash content (wt%) was measured by a standard gravimetric ashing procedure (700 °C, 2 h).

Nitrogen adsorption–desorption analysis (ASAP 2020, Micromeritics, Norcross, GA, USA) was conducted at 77 K to determine the specific surface area and porosity. The Brunauer–Emmett–Teller (BET) method was applied to calculate the surface area, and micropore parameters were obtained using the Dubinin–Astakhov model. The BET surface area was calculated from adsorption data in the relative pressure range (P/P0 = 0.05–0.30).

Biochar and BBCs for characterization were prepared from at least three independent batches. BET analyses were carried out in triplicate, and the reported parameters represent mean values (*n* = 3). SEM and XPS data are presented as representative images from randomly selected regions.

### 4.2. TCC Removal Experiment

Four experimental groups were established: *P. chlororaphis* (PC), corn straw biochar + *P. chlororaphis* (CS + PC), corn cob biochar + *P. chlororaphis* (CC + PC), and rice husk biochar + *P. chlororaphis* (RH + PC). Three abiotic controls without bacterial inoculation (CS, CC, and RH) were also prepared.

To ensure TCC served as the sole carbon source, BBCs and a TCC solution were added to 40 mL of M9 medium lacking glucose, giving an initial TCC concentration of 1 mg/L and a carrier suspension concentration of 1 g/L. Incubations were performed at a setpoint of 30 °C, which was maintained throughout the degradation process, and 160 rpm in a shaking incubator for 48 h, with an initial pH of 7.4. A 48 h incubation period was selected as the endpoint based on preliminary tests showing that bacterial growth and TCC removal approached a steady state after 2 days. After incubation, the whole slurry (culture medium together with suspended biochar, where applicable) was extracted three times with dichloromethane to quantify total residual TCC (aqueous + sorbed), and the solvent was subsequently replaced with methanol. The residual TCC concentration was determined using high-performance liquid chromatography (HPLC, LC-20AR HPLC system, Shimadzu, Kyoto, Japan). The mobile phase consisted of acetonitrile and water (80:20, *v*/*v*) at a flow rate of 1.0 mL/min; the detection wavelength was 275 nm, and the injection volume was 20 µL. This method quantifies residual parent TCC after whole-slurry extraction; it does not resolve transformation products and therefore does not by itself demonstrate complete mineralization.

Method recovery was evaluated by spike-recovery tests at 1 mg L^−1^ (*n* = 3) following the same extraction and analysis procedure, giving recoveries of 95.8 ± 3.1% for the PC matrix and 94.7 ± 1.5% for the biochar-containing matrix (mean ± SD). Because whole-slurry extraction was used, adsorption to biochar was not subtracted in the calculation of removal.

Removal (%) was calculated as

(1) $$Removal\;(\%)=\left(1-\frac{C_{TCC}}{C_{0}}\right)\times 100\%$$

where *C_TCC_* is the residual TCC concentration (mg/L), and *C*_0_ represents the initial TCC concentration (mg/L).

Each treatment group and control group were set up with four parallel replicates (*n* = 4).

Accordingly, the present endpoint should be interpreted as removal of the parent compound, and it does not quantify dechlorination (chloride release) or toxicity changes of the reaction mixture.

### 4.3. FTIR Identification of Functional Groups

FTIR analysis was performed to examine changes in the surface chemical structures of biochars, bacterial cells, and their composites, thereby elucidating the behavior of electron-transfer-related functional groups during TCC interaction. Seven sample groups were prepared: PC, CS, CC, RH, CS + PC, CC + PC, and RH + PC. Each group was also analyzed after exposure to 1 mg/L TCC for 48 h. After freeze-drying, samples were mixed with KBr and pressed into pellets. FTIR spectra were collected in the range of 500–4000 cm^−1^ at a resolution of 4 cm^−1^ with 32 scans per sample. Variations in the intensity and position of characteristic peaks—including hydroxyl (O-H), carboxyl (-COOH), amide (-CONH-), carbonyl (C=O), and aromatic C=C—were compared to identify functional-group transformations induced by TCC and to determine key chemical sites involved in EET and interfacial interactions. FTIR spectra were recorded using a Vertex 70 FTIR spectrometer (Bruker, Ettlingen, Germany).

FTIR measurements were repeated to confirm spectral stability, and representative spectra are presented for each sample group.

### 4.4. Electrochemical Analysis of Biochar–Bacteria Composites

Electrochemical analyses were performed using a CHI 660E workstation (Chenhua, Shanghai, China) to evaluate the electron-transfer behavior of biochar, bacterial cells, and their composites after exposure to 1 mg/L TCC. Measurements included cyclic voltammetry (CV) and electrochemical impedance spectroscopy (EIS). Seven sample groups were tested: PC, CS, CC, RH, CS + PC, CC + PC, and RH + PC.

For sample preparation, 8 mg of each material was dispersed in 1 mL of an ethanol/water mixture (1:4, *v*/*v*) containing 40 µL of Nafion solution. The suspension was vortexed until homogeneous, and 100 µL was drop-cast onto a 1 cm^2^ indium tin oxide (ITO) conductive substrate, then air-dried for testing.

EIS and CV measurements were performed in a three-electrode configuration using the sample-loaded ITO conductive glass as the working electrode, a saturated calomel electrode (SCE) as the reference electrode, and a platinum foil as the counter electrode. The electrolyte consisted of 0.1 mol/L KCl, which was purged with nitrogen for 10 min before measurement to remove dissolved oxygen. CV scans were recorded from −0.8 to 1.0 V at a scan rate of 50 mV/s.

These measurements provided descriptors such as Rct and Zw, redox peak currents for comparing electron-transfer behavior among samples.

For each sample group, EIS and CV measurements were repeated to verify reproducibility, and representative Nyquist plots and cyclic voltammograms are shown.

### 4.5. Non-Targeted Metabolomic Analysis Based on LC-MS

To explore how different biochar carriers influence the reconfiguration of metabolic pathways in *P. chlororaphis*, a non-targeted metabolomic analysis was performed using liquid chromatography–mass spectrometry (LC-MS, Thermo Fisher Scientific, UHPLC-Exploris240, Waltham, MA, USA). Four experimental groups were analyzed: PC, CS + PC, CC + PC, and RH + PC. Each experimental group was analyzed with six biological replicates (*n* = 6). To ensure analytical reproducibility, 3 technical replicates were performed for each biological sample. Metabolites were extracted from the cell-free supernatant and analyzed on a Thermo Scientific UHPLC-FTMS system. The data were processed using Progenesis QI and statistically evaluated with MetaboAnalyst5.0 and R (v4.3.0). Principal component analysis (PCA) and variable-importance projection (VIP) values were used to identify significantly altered metabolites (VIP > 1, *p* < 0.05). For multi-group comparisons, one-way ANOVA was employed, and *p*-values obtained from enrichment analyses (DA-score and KEGG pathway enrichment) were adjusted using the Benjamini–Hochberg method to account for multiple testing. The potential metabolic pathways affected by biochar loading were identified through pathway enrichment analysis using the KEGG database (https://www.kegg.jp/).

Detailed procedures for sample preparation, LC-MS conditions, quality control, and raw-data processing are provided in the Supporting Information (Text S1).

## 5. Conclusions

This study supports a feedstock-dependent, multilayer interpretation linking the biochar structure, interfacial chemistry, electrochemical behavior and extracellular metabolomic signatures to enhance TCC biodegradation by biochar-immobilized *Pseudomonas chlororaphis*. Across the tested systems, biochar loading was associated with higher TCC removal, reproducible interfacial functional-group perturbations (FTIR), and improved interfacial charge-transfer kinetics (CV/EIS; Rct), together with statistically supported extracellular metabolomic remodeling. Collectively these observations are consistent with a coupled “structure–interface–metabolism” framework in which interfacial redox-active functionalities and redox-related metabolic signatures may contribute to EET and pollutant transformation. Among feedstocks, corn cob biochar was associated with stronger quinone-/aromatic-intermediate signatures consistent with higher electron–donor potential and better suitability for high-load polishing of halogenated micropollutants; corn straw biochar showed mediator/transport-related features suggestive of sustained electron exchange; and rice husk biochar showed PPP/GSH-associated signatures suggestive of enhanced redox-maintenance/regeneration capacity. Because quinone-like moiety densities and intracellular redox couples were not directly quantified, these mechanistic attributions should be regarded as evidence-supported interpretations that motivate targeted validation. Future work will evaluate these composites in continuous-flow tests with real wastewater and couple performance assessments to support scale-up.

## Figures and Tables

**Table 1:** Physicochemical characteristics of CS, CC and RH biochars, including BET surface area, total pore volume, average pore diameter, elemental composition and pH. BET parameters represent mean values from triplicate measurements (*n* = 3).

Sample	BET Surface Area (m^2^/g)	Total Pore Volume (cm^3^/g)	Average Pore Diameter (nm)	C (wt%)	O (wt%)	H (wt%)	N (wt%)	Ash (wt%)	pH
CS	85.51	3.80	139.68	83.00	9.95	1.03	1.42	4.60	9.46
CC	12.96	0.60	14.96	83.44	11.93	1.03	1.30	2.30	8.24
RH	52.30	0.04	88.65	53.90	33.30	1.21	0.92	10.67	9.86

Elemental contents are reported on a dry basis (wt%). Oxygen was calculated by difference: O (wt%) = 100 − C–H–N − ash.

**Table 2:** EIS fitting parameters for PC, biochars (CS, CC, RH) and their composites (CS + PC, CC + PC, RH + PC). Solution resistance (Rs), charge transfer resistance (Rct) and Warburg impedance (Zw) are shown as representative fitted values from electrochemical impedance spectra.

Sample	Rs (Ω)	Rct (Ω)	Zw (Ω)
CS + PC	3.82 × 10^1^	9.65 × 10^4^	9.35 × 10^−4^
CS	4.12 × 10^1^	4.94 × 10^4^	2.60 × 10^−5^
CC + PC	3.71 × 10^1^	1.76 × 10^5^	1.08 × 10^−3^
CC	3.07 × 10^1^	1.38 × 10^4^	3.64 × 10^−4^
RH + PC	3.83 × 10^1^	5.64 × 10^3^	9.78 × 10^−4^
RH	3.89 × 10^1^	5.12 × 10^4^	6.36 × 10^−4^
PC	3.37 × 10^1^	2.54 × 10^5^	6.19 × 10^−5^

Table notes: All values are shown in scientific notation. Rs: solution resistance; Rct: charge transfer resistance; Zw: Warburg impedance.

## Data Availability

The original contributions presented in this study are included in the article/supplementary material. Further inquiries can be directed to the corresponding author.

## Supplementary Materials

The following supporting information can be downloaded at https://www.mdpi.com/article/10.3390/ijms27062684/s1.

## Abbreviations

The following abbreviations are used in this manuscript:

BBCs	Biochar–bacteria composites
CC	Corn cob
CEC	Contaminant of emerging concern
CS	Corn straw
CV	Cyclic voltammetry
EET	Extracellular electron transfer
EIS	Electrochemical impedance spectroscopy
PCA	Principal component analysis
POPs	Persistent organic pollutants
RH	Rice husk
TCC	Triclocarban
SCE	Saturated calomel electrode
