# Supplementary material for: Tailored Biochar–Pseudomonas chlororaphis Composites for Triclocarban Removal: A Feedstock-Dependent Structure–Interface–Metabolism Study
Source: Int J Mol Sci. 2026 Mar 15;27(6):2684. doi: 10.3390/ijms27062684 (PMC13027295; doi:10.3390/ijms27062684)
Supplement: Supplementary file 1 [file ijms-27-02684-s001.zip › ijms-4160391-supplementary.pdf]

Supplementary Material for

Tailored Biochar–Pseudomonas chlororaphis Composites for Triclocarban Removal: A Feedstock-Dependent Structure–Interface–Metabolism Study

Text S1. Detailed Procedure for LC-MS-Based Non-Targeted Metabolomics

Sample Processing and Metabolite Extraction: After the reaction, samples from each group were immediately collected and processed at low temperature to maintain metabolite stability. The culture supernatants were freeze-dried, and the resulting samples were extracted using a precooled methanol-water mixture (4:1, v/v). Four internal standards (e.g., L-2-chlorophenylalanine, 0.02 mg/mL) were added to the extraction solvent for subsequent signal stability correction. For each sample, 20 mg of freeze-dried material was weighed into a 2 mL centrifuge tube containing one 6 mm stainless-steel bead. The samples were ground at −10 °C and 50 Hz for 6 min using a cryogenic tissue grinder, followed by ultrasonication at 5 °C and 40 kHz for 30 min in a temperature-controlled ultrasonic cleaner. The extracts were then left to stand at −20 °C for 30 min to promote protein precipitation. After centrifugation (13 000 × g, 4 °C, 15 min), the supernatants were transferred to autosampler vials with inserts for LC-MS analysis. For quality control (QC), 20 μL of supernatant from each sample was pooled to prepare a composite QC sample, which was used to assess the stability of the detection system.

LC-MS Conditions: Metabolite detection was performed using a Thermo Scientific UHPLC-Exploris 240 ultra-high-performance liquid chromatography-Fourier transform mass spectrometry (UHPLC-FTMS) system. The analytical column was an ACQUITY UPLC HSS

T3 (100 mm × 2.1 mm, 1.8 μm; Waters, USA). The column temperature was maintained at 40 °C, and the injection volume was 3 μL.The mobile phase consisted of solvent A (95% water + 5% acetonitrile containing 0.1% formic acid) and solvent B (47.5% acetonitrile + 47.5% isopropanol + 5% water containing 0.1% formic acid). A gradient elution program was used to achieve multicomponent separation.An electrospray ionization (ESI) source was applied, and data were acquired in both positive and negative ion modes. The detailed parameters were as follows: scan range, *m/z* 70-1050; sheath gas flow rate, 60 arb; auxiliary gas flow rate, 20 arb; heater temperature, 350 °C; capillary temperature, 320 °C; spray voltage, +3400 V (positive mode) and -3000 V (negative mode); S Lens RF Level, 70; normalized collision energy, 20/40/60%; full-scan resolution, 60 000; MS^2 resolution, 15 000.

Quality Control and Data Processing: One QC sample was inserted after every five analytical samples to monitor instrument drift and evaluate system stability in real time. The acquired raw data were processed using Progenesis QI v3.0 (Waters Corporation, USA) for baseline correction, peak extraction, alignment, and integration, generating a data matrix containing retention time, mass-to-charge ratio (*m/z*), and peak intensity information.Feature ions were annotated by matching MS and MS/MS spectra with public databases, including HMDB (http://www.hmdb.ca/), METLIN (https://metlin.scripps.edu/), and an in-house metabolite library, with a mass error tolerance within 10 ppm. Normalized and internal-standard-corrected data were imported into MetaboAnalyst 5.0 and R (v4.3.0) for multivariate statistical analyses, including principal component analysis (PCA) and differential metabolite screening (VIP > 1, $p < 0.05$).Significantly altered metabolites were further subjected to pathway enrichment analysis in the KEGG database (https://www.kegg.jp/) to elucidate the potential link between EET and metabolic reprogramming of microorganisms under biochar-loading conditions.
